# Supramalleolar Osteotomy for Varus Ankle Osteoarthritis: A Contemporary Narrative Review

**DOI:** 10.3390/medicina62101983

**Published:** 2026-10-14

**Authors:** Silvio Caravelli, Marco Di Ponte, Luca Berveglieri, Giammarco Gardini, Hassan Zmerly, Edoardo Cassanelli, Emanuele Vocale, Stefano Zaffagnini, Massimiliano Mosca

**Affiliations:** 1 U.O. Ortopedia Bentivoglio, IRCCS Istituto Ortopedico Rizzoli, 40136 Bologna, Italy; 2 Department of Biomedical and Neuromator Sciences (DIBINEM), University of Bologna, 40126 Bologna, Italy; 3 Department of Life Sciences, Health, and Health Professions, Link Campus University, 00165 Rome, Italy; 4 Clinica Ortopedica e Traumatologica II, IRCCS Istituto Ortopedico Rizzoli, 40136 Bologna, Italy

**Keywords:** SMOT, total ankle replacement, ankle arthrodesis, CORA, malalignment, treatment algorithm, surgical treatment

## Abstract

*Background and Objectives:* Varus ankle osteoarthritis (OA) is most commonly post-traumatic and is characterized by asymmetric degeneration and medial overload of the tibiotalar joint. Supramalleolar osteotomy (SMOT) is a joint-preserving procedure that aims to restore mechanical alignment, redistribute joint contact forces, and delay, or potentially avoid, total ankle replacement (TAR) or ankle arthrodesis (AA). However, the optimal choice of technique and amount of correction remain debated, particularly in complex multiplanar deformities. *Materials and Methods:* This narrative review summarizes current evidence on SMOT for varus ankle OA. Relevant clinical, radiographic, biomechanical, imaging, and surgical studies were selected and synthesized narratively, with particular emphasis on deformity classification, location of the center of rotation of angulation (CORA), joint congruency, surgical technique, clinical outcomes, complications, and treatment planning. *Results:* SMOT provides reliable pain relief and functional improvement in patients with Takakura–Tanaka stage 2–3A ankle OA, with reported mid-term survival rates of 88–93%. The choice of technique should be guided by the location of the deformity, the degree of talar tilt, and overall limb alignment. In selected cases, a concomitant fibular osteotomy improves talar reduction and medial unloading. Three-dimensional planning and patient-specific instrumentation may improve the accuracy of correction. *Conclusions:* SMOT is an effective joint-preserving treatment for varus ankle OA, provided that the deformity is accurately analyzed and patients are carefully selected. This review proposes a simplified, stepwise treatment algorithm, supported by illustrations, to help surgeons select the most appropriate osteotomy technique according to the deformity pattern.

## 1. Introduction

Osteoarthritis is a leading cause of pain and disability worldwide. In 2020, an estimated 595 million people, or 7.6% of the global population, were living with OA [1]. Unlike hip and knee OA, ankle OA is most frequently post-traumatic: approximately 78% of cases result from previous fractures or ligament injuries, whereas only 9% are primary. The remaining cases are secondary to systemic conditions (e.g., rheumatologic diseases, hemophilia) or local pathologies (e.g., osteomyelitis, avascular necrosis, osteochondritis dissecans) [2].

Surgical treatment of ankle OA can be divided into joint-sparing and joint-sacrificing procedures. Joint-sparing procedures have gained popularity for young, active patients with moderate ankle OA, in whom TAR or AA may not be indicated. These include arthroscopic or open joint debridement, distraction arthroplasty, osteochondral resurfacing, and corrective osteotomies [3]. Osteotomies are based on the principle that ankle pain and joint degeneration arise from underlying or post-traumatic malalignment [3]. Joint-preserving procedures aim to relieve symptoms, reduce harmful joint loading, and slow or halt the degenerative process [4].

Current controversies regarding SMOT include the optimal amount of correction, the role of a concomitant fibular osteotomy, and the long-term implications for subsequent ankle arthroplasty. The main clinical challenges are accurate patient selection, avoidance of overcorrection, and management of multiplanar deformities.

## 2. Background

A lateral shift of the talus of only 1 mm has been reported to reduce the tibiotalar contact area by up to 42%, illustrating how sensitive the ankle is to malalignment [5].

Ankles with pathological varus deformity are characterized by progressive medial overload of the tibiotalar joint. The ability of the foot and ankle to tolerate malalignment depends largely on the compensatory flexibility of the adjacent joints. In particular, the subtalar joint may evert to maintain a plantigrade foot when a proximal varus deformity is present. Experimental studies have shown that, in the early stages of varus malalignment, the center of force may shift laterally, increasing lateral stress. As the deformity progresses, however, this compensation becomes insufficient: the center of force migrates medially and lateral stress decreases. This pattern probably reflects an initial valgus tilt of the subtalar joint as it attempts to realign the foot, followed by decompensation and loss of normal biomechanics. The primary goal of realignment surgery is therefore to restore a more physiological load distribution across the ankle by unloading the degenerated compartment. A well-performed SMOT has been reported to delay the need for TAR by approximately 5 years with good results and, in some cases, may be the definitive treatment, avoiding further surgery [6].

SMOT is generally indicated in patients with asymmetric varus ankle OA in whom a substantial portion of the tibiotalar joint surface is preserved, most commonly corresponding to Takakura–Tanaka stages 2–3A. The Takakura–Tanaka classification grades ankle OA according to the location and extent of joint space narrowing and subchondral bone involvement, ranging from early obliteration of the medial joint space (stage 2) to complete bone-on-bone contact and advanced joint destruction (stage 4) [7]. Stage 3A, in which a substantial portion of the joint surface is still preserved, is generally regarded as a potential indication for joint-preserving procedures such as SMOT. However, the amount of preserved cartilage should be interpreted together with joint congruency, severity of deformity, symptoms, and overall limb alignment.

Although SMOT may delay joint-sacrificing surgery in selected patients, progression of OA or failure of the procedure may eventually require conversion to TAR or AA. In a systematic review of 11 studies including 657 SMOTs, conversion to TAR and AA was reported in 5.8% and 2.7% of cases, respectively, at mean intervals of 37 and 45 months after the index procedure [8]. The literature reports an overall complication rate of 5.1%, a failure rate of 6.8%, and a mean survival rate of 88–93% for ankles with preoperative Takakura–Tanaka stage 2 or 3A, with only 12% requiring TAR or AA [6,9,10]. A Takakura–Tanaka stage of 3B or higher appears to worsen the prognosis, possibly because the overall deforming forces are too high to be fully corrected by SMOT alone [4,11]. Despite these favorable results, determining the exact amount of correction required and selecting the most appropriate technique for each patient remain challenging.

This review aims to provide a practical, clinically oriented discussion of SMOT for varus ankle OA. The available literature was reviewed with regard to the main aspects of patient assessment and surgical management, including deformity location, joint congruency, radiographic evaluation, biomechanics, surgical techniques, clinical outcomes, and complications, and the evidence was interpreted narratively to support the proposed treatment considerations. As this is a narrative review, no formal systematic review protocol, predefined screening process, risk-of-bias assessment, structured data extraction, or meta-analysis was performed. In particular, we describe the main SMOT techniques used for the different patterns of varus deformity and propose a simplified treatment algorithm, supported by schematic illustrations of each osteotomy to facilitate understanding.

## 3. Presentation

### 3.1. Indications and Clinical Evaluation

The choice of treatment for each patient depends on the analysis of etiological risk factors and comorbidities. The main indications and contraindications for SMOT are summarized in Table 1.

Patient selection should consider the stage of OA, the amount of preserved joint surface, the alignment and stability of the ankle and hindfoot, bone quality, soft-tissue condition, and medical comorbidities. End-stage OA, active infection, severe vascular or neurological impairment, and uncontrollable ankle instability generally preclude joint-preserving realignment surgery. Conversely, Takakura–Tanaka stage 3B, older age, smoking, diabetes mellitus, osteoporosis, and poor bone quality should be regarded as relative contraindications or as factors requiring individual assessment [4,12]. The role of age remains highly debated. An age over 60 years is generally considered a relative contraindication to SMOT; however, favorable outcomes have also been reported in older patients, provided that thorough preoperative planning is performed and accurate correction is achieved [13].

Overall alignment is assessed with the patient standing, and the deformity is then reassessed with the patient on tiptoe to determine whether the eccentric pull of the Achilles tendon influences the hindfoot deformity. Patients with ankle varus may have compensatory hindfoot valgus; in these cases, correcting the ankle varus may result in hindfoot valgus. The Coleman block test is performed to identify a forefoot-driven deformity. In varus deformities, hindfoot alignment makes it difficult to identify the apex of the deformity accurately. Varus ankle deformity is often associated with lateral ankle instability, which causes a medial shift of the talar center of rotation. This displaces the load-bearing axis, overloads the medial ankle, and leads to medial malleolar dysplasia, resulting in a complex intra-articular deformity [14,15]. In addition, with long-standing chronic instability, the peroneal tendons are often torn, and the posterior and anterior tibial tendons further exacerbate the varus deformity of the foot [16,17]. Concomitant sagittal plane deformities, such as procurvatum or recurvatum, may also be present. Procurvatum is generally less well tolerated because of anterior impingement, although it protects against joint degeneration by providing greater talar coverage [18].

With the patient seated, palpation around the joint identifies areas of tenderness. Active and passive ankle range of motion, as well as subtalar and midfoot motion, are measured, and medial and lateral ankle stability is assessed. Limb-length discrepancy should also be measured, as it may influence the choice of osteotomy. Finally, the function and strength of the posterior tibial tendon (inversion) and the peroneus brevis tendon (eversion) are evaluated. Varus ankle OA typically presents with deep ankle pain, swelling, and reduced dorsiflexion. Patients may also report instability, particularly on uneven ground.

### 3.2. Radiographic Evaluation

The surgeon must identify the CORA when analyzing the deformity and planning its correction. The CORA is the apex of the deformity. The closer the CORA is to the ankle joint, the greater its effect on ankle malrotation and on the medial distal tibial angle [4]. In many cases, the apex lies very close to, or even within, the joint. The osteotomy should be performed at the level of the CORA to minimize translation and rotation of the distal segment. When varus is corrected, an osteotomy with a wedge proximal to the CORA results in lateralization of the weight-bearing axis [19].

On weight-bearing anteroposterior ankle radiographs, distal tibial alignment can be assessed using the tibial articular surface (TAS) angle, also referred to as the medial distal tibial angle (MDTA). This angle is formed by the tibial mechanical axis and a line connecting the medial and lateral margins of the tibial plafond; its reported normal value is approximately 93.3 ± 3.2° [20]. The tibiotalar surface (TTS) angle is measured between the tibial mechanical axis and the superior articular surface of the talar dome and is approximately 87.2 ± 2.8° in normally aligned ankles [21]. Talar tilt (TT) reflects the angular incongruence between the tibial plafond and the talar dome; when the TAS and TTS angles are measured on the same weight-bearing radiograph, TT corresponds to their absolute difference (TT = |TAS − TTS|). As the reference values reported above derive from different cohorts, they should not be subtracted from each other to obtain a normal TT value. A TT below 4° is generally consistent with a congruent ankle, whereas higher values indicate tibiotalar incongruence. These parameters can help distinguish predominantly supramalleolar, intra-articular, and mixed deformity patterns (Table 2); however, the reported thresholds should be interpreted in conjunction with weight-bearing imaging, deformity location, and overall limb alignment. Any sagittal plane deformity should also be noted. The Saltzman hindfoot alignment view can help detect subtalar malalignment [22].

The height (H) of the wedge to be removed or inserted (for closing- or opening-wedge osteotomies, respectively) can be calculated as H = tan α × W, where α is the required angle of correction and W is the width of the distal tibia, corrected for magnification using a radiopaque marker of known size [23]. The MDTA is usually overcorrected intraoperatively by 2–3° to avoid a paradoxical increase in talar tilt after SMOT [24]. Although this principle is widely applied, there is no consensus on the optimal amount of MDTA correction after valgus SMOT [25,26]. Conventional radiographs, however, are prone to measurement errors in assessing ankle alignment because of variations in patient positioning, particularly limb rotation and beam centering [4]. In addition, alignment measurements differ depending on whether short or long tibial radiographs are used [20]. A further limitation of plain radiographs is that they provide a two-dimensional (2D) projection of a deformity that is often three-dimensional (3D) [27].

Proper planning of SMOT requires a thorough understanding of overall lower-limb alignment, as ankle deformities are frequently associated with proximal malalignment, particularly at the knee and distal femur. Recent studies have shown that, in patients with ankle OA, hindfoot varus or valgus is often accompanied by compensatory valgus or varus alignment of the knee, respectively, suggesting a correlated deviation of the mechanical axis along the entire limb [28,29]. Preoperative evaluation should therefore include full-length weight-bearing radiographs of the lower limbs [30,31].

## 4. Treatment

### 4.1. Transverse Medial Opening-Wedge Osteotomy

Medial opening-wedge SMOT allows controlled correction of the deformity through progressive distraction of the osteotomy. The size of the wedge is estimated during preoperative planning and can be refined using intraoperative imaging. Asymmetric distraction at the osteotomy site allows correction in both the coronal and sagittal planes, depending on the pivot point of distraction.

**Figure 1:**
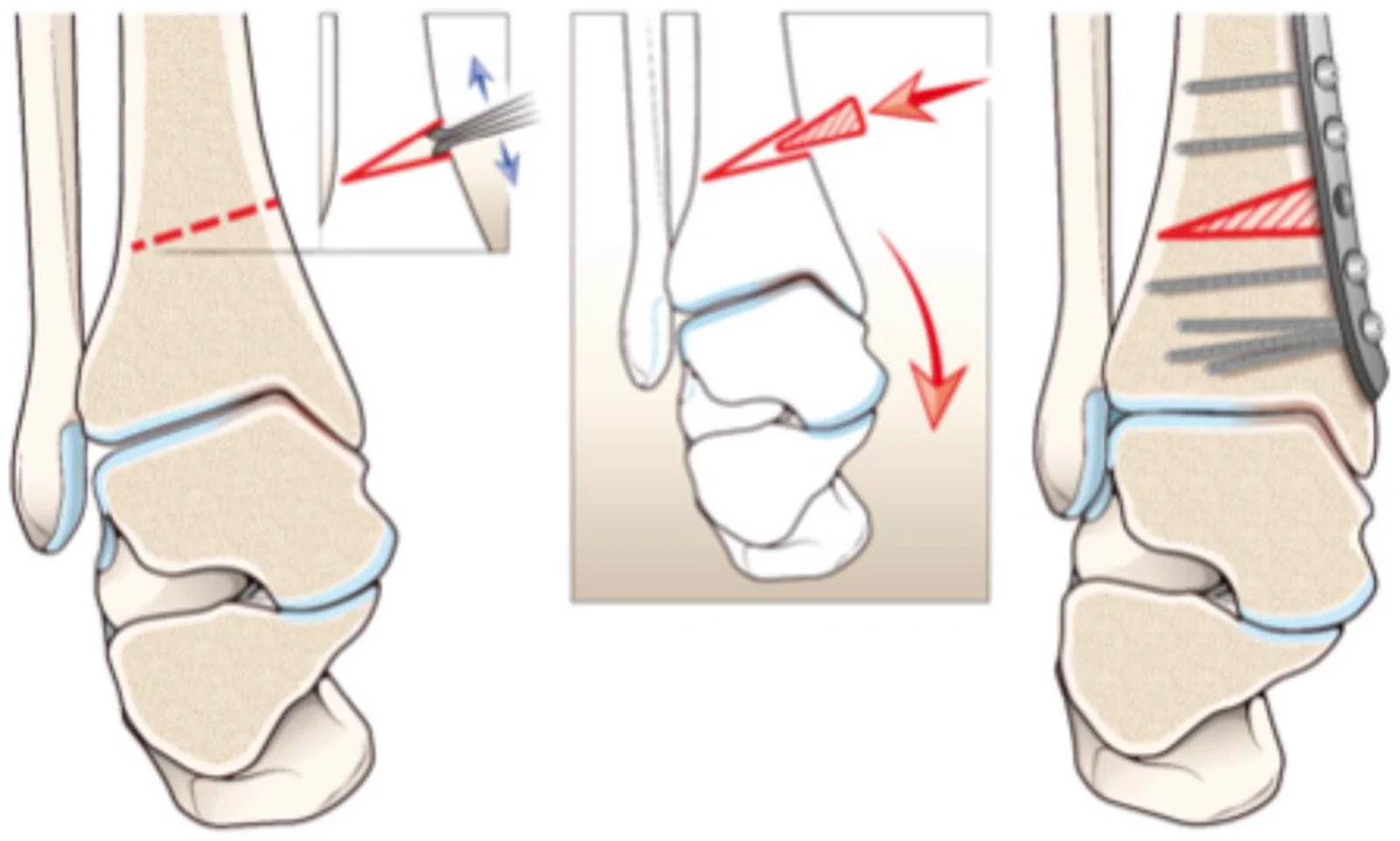
Illustration of transverse medial opening-wedge osteotomy with bone graft (red arrow) for the correction of non-congruent ankle varus deformity.

A medial approach centered on the medial malleolus is typically used. The level of the osteotomy is identified under fluoroscopy, and two parallel K-wires are placed at this level. The osteotomy is started with an oscillating saw and completed with an osteotome to minimize the risk of breaching the lateral cortex, which would compromise the stability of the osteotomy. If the lateral cortex is breached, additional lateral fixation through a separate approach is advisable. The osteotomy is then opened with a distractor (e.g., the Hintermann distractor) and fixed with dedicated anatomical plates (Figure 1).

For larger corrections, particularly when the opening exceeds approximately 6°, a structural graft may be considered; however, the need for grafting and the choice of graft depend on the size and geometry of the gap, bone quality, osteotomy stability, and the fixation construct [32]. A concomitant fibular osteotomy is also recommended, as it allows a greater shift of the talus and thus a more effective redistribution of contact pressure across the ankle joint. Clinically, this results in better correction of both talar tilt and the tibiocrural angle [33].

Choi et al. recently reported the mid- to long-term outcomes of 42 ankles (39 patients) treated with medial opening-wedge SMOT, with a mean follow-up of 106.2 ± 8.8 months [34]. Radiographic improvement was observed in 71.4% of Takakura–Tanaka stage 2 and 85.0% of stage 3A ankles, whereas 73.3% of stage 3B ankles remained unchanged. Clinical scores improved significantly across the entire cohort; in stage 3B ankles, the mean AOFAS score increased from 43.5 to 82.4 in those with radiographic improvement and from 41.1 to 73.2 in those without. Failure was associated with excessive valgization of the tibial plafond: with the talus remaining in varus, this produced a paradoxical increase in talar tilt, and stage 3B ankles that improved had a significantly smaller medial opening (7.4 vs. 11.3 mm). Five ankles (11.9%) were ultimately converted to ankle arthrodesis.

### 4.2. Lateral Closing-Wedge Osteotomy

For larger coronal deformities, typically 10° or more, a lateral closing-wedge osteotomy may be considered, particularly when a medial opening wedge would require a large gap or place excessive tension on the medial soft tissues [22]. Its advantages include greater stability, no need for bone graft, the possibility of performing the fibular osteotomy through the same incision, and greater correction of talar tilt. Its limitations include the inability to adjust the correction gradually, the need for a biplanar cut when a concomitant sagittal deformity must be corrected, limb shortening, and weakening of the lateral compartment muscles [4].

**Figure 2:**
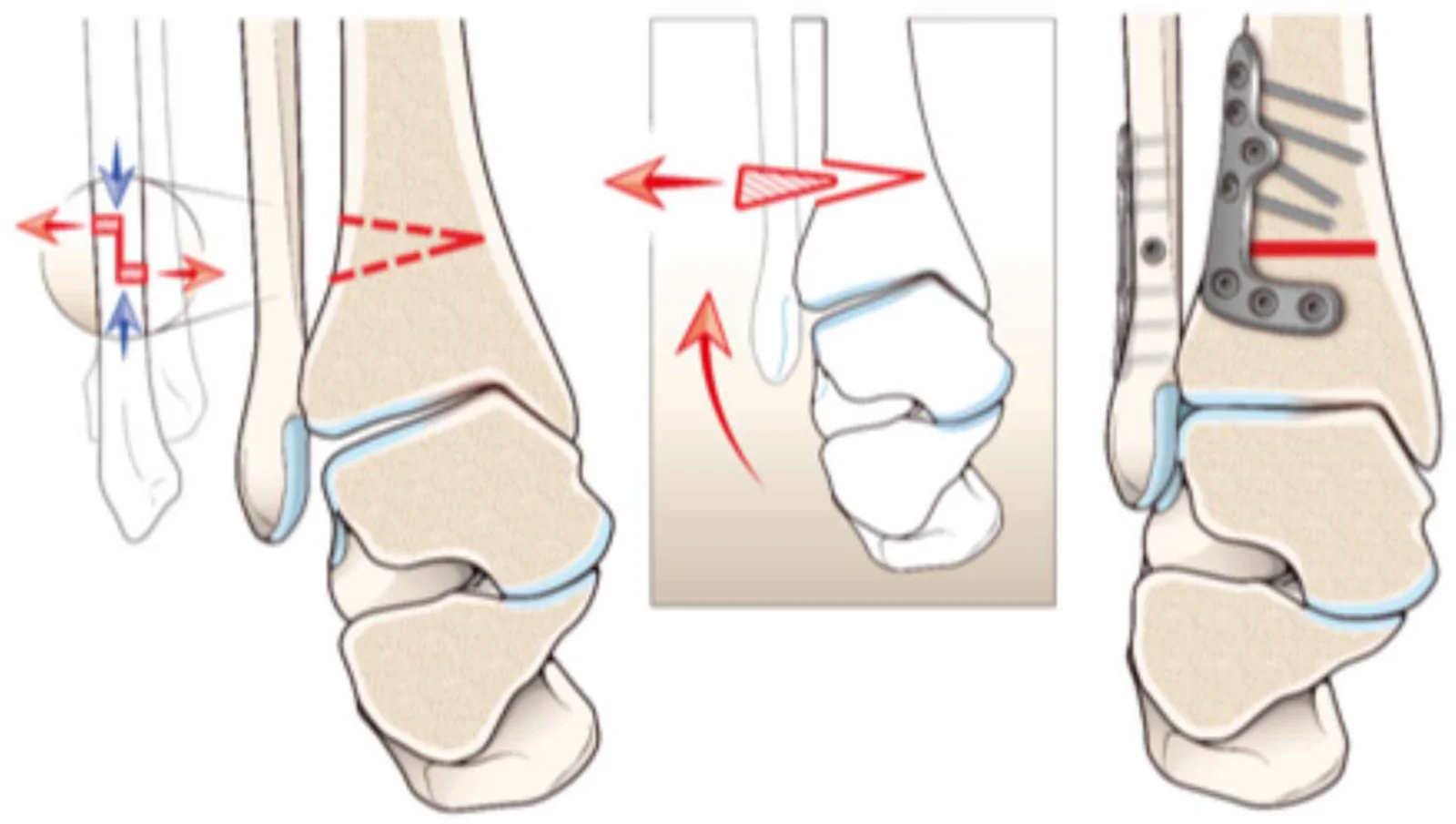
Illustration of lateral closing wedge with bone removal (red arrow) and with fibular osteotomy for correction of non-congruent ankle varus deformity.

A direct lateral approach to the fibula is used, with the incision extending proximally from the tip of the fibula along its distal third [35]; the syndesmosis is also exposed through this approach. A shortening osteotomy of the fibula is required to achieve better correction and joint congruency. A Z-shaped osteotomy is inherently more stable than a transverse one and reduces the risk of fibular malposition [19]. Under fluoroscopy, two K-wires are inserted into the tibia according to the preoperative plan to guide the cut. The closing-wedge osteotomy is then performed with an oscillating saw and completed with an osteotome, taking care to preserve the medial cortex. The tibial osteotomy is fixed with an L-shaped anterolateral plate, and the Z-shaped fibular osteotomy is fixed in the shortened position with an interfragmentary screw and a neutralization plate (Figure 2).

Harstall et al. evaluated nine patients treated with lateral closing-wedge osteotomy for varus ankle deformity, with a mean follow-up of 56 months (range, 15–88) [35]. The AOFAS score increased significantly from 48 ± 16 preoperatively to 74 ± 11.7 postoperatively, and the mean TAS angle improved from 6.9 ± 3.8° of varus to 0.6 ± 1.9° of valgus, with no changes in the sagittal plane.

### 4.3. Oblique Distal Tibial Osteotomy

The ankle mortise is essential for joint stability, and its loss may accelerate degenerative changes. In advanced medial ankle OA, the mortise often widens as the medially displaced talus erodes the medial side. This widening of the medial gutter further reduces joint stability and contributes to the progression of degeneration. Transverse supramalleolar osteotomies, whether opening or closing, can correct coronal and sagittal deformities but do not address the width of the mortise.

**Figure 3:**
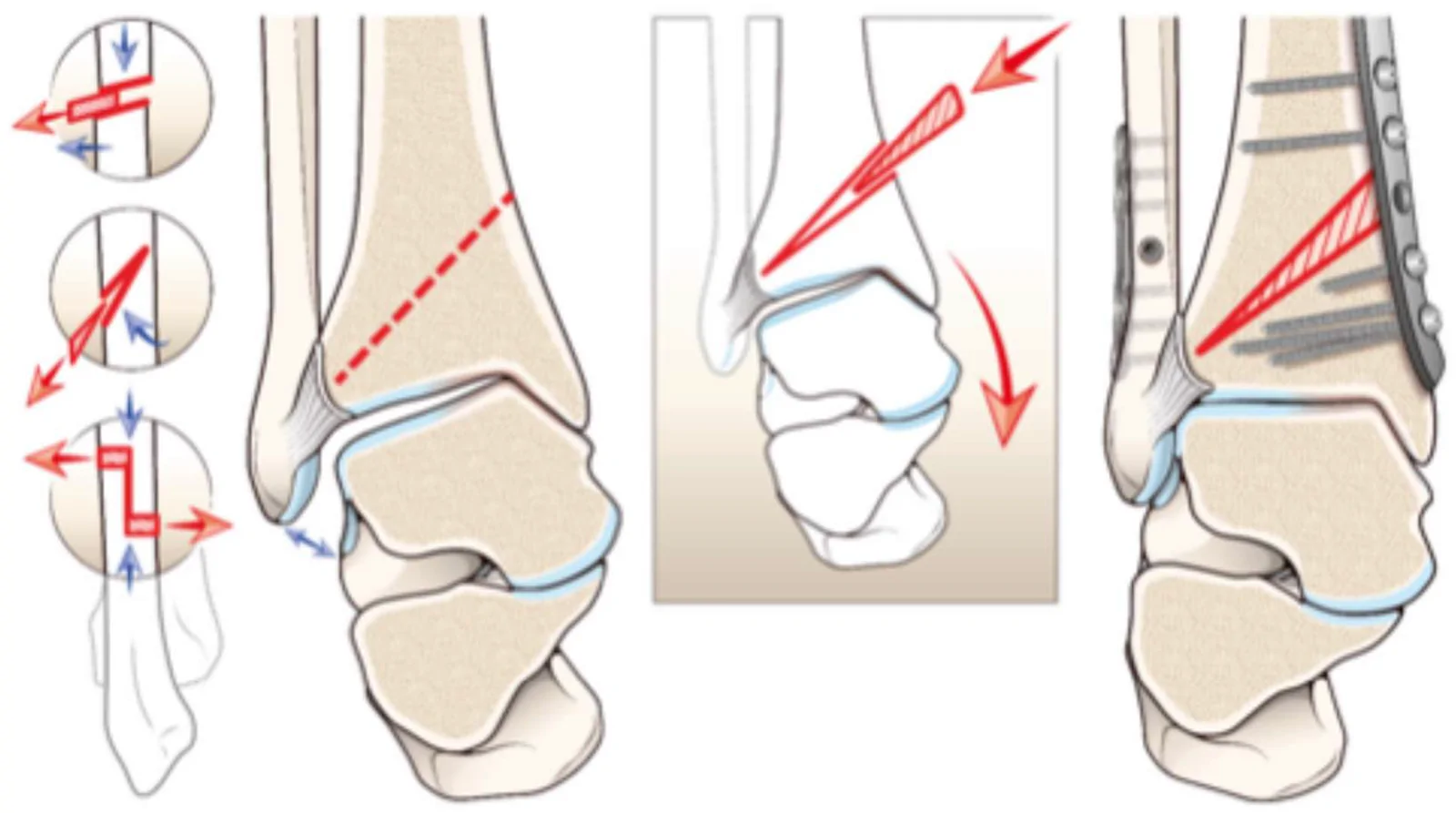
Illustration of oblique medial opening-wedge with bone graft (red arrow) and with fibular osteotomy for the correction of non-congruent ankle varus deformity.

An oblique distal tibial osteotomy performed without a fibular osteotomy can simultaneously correct the coronal varus deformity and narrow the ankle mortise [36]. Through an anterior approach, the osteotomy line is planned from a medial point approximately 5 cm proximal to the joint line to a lateral point approximately 5 mm proximal to the joint line. Two K-wires are inserted under fluoroscopic guidance to guide the osteotomy, which is prepared by drilling along the planned line and completed by connecting the drill holes with an osteotome. The distal fragment is then distracted and rotated, correcting the deformity and closing the mortise (Figure 3), and is fixed with a locking plate.

Ahn et al. reported excellent radiographic and clinical outcomes in a cohort of 18 patients treated with oblique distal tibial osteotomy without fibular osteotomy [36]. The MDTA was significantly corrected, from a mean of 86.6° to 92.9°, and the ADTA and AOFAS score also improved, confirming the effectiveness of this technique in restoring coronal alignment and ankle stability while preserving the joint.

Unlike conventional SMOTs, the oblique distal tibial osteotomy alters the inclination of the tibial plafond and closes the mortise, and may therefore increase talar tilt. It may be more suitable when preoperative talar tilt is limited, as some authors have reported less predictable results when the tilt exceeds approximately 5–7° [32]. Adding a fibular osteotomy to the oblique distal tibial osteotomy has been shown to improve the alignment of the talus within the mortise.

Liang et al. analyzed 38 patients with varus ankle OA, divided into two groups according to whether a concomitant fibular osteotomy was performed [37]. In the fibular osteotomy group (n = 24), medial talar displacement and talar tilt improved significantly more, with talar tilt decreasing from 6.3 ± 2.2° to 2.6 ± 1.1° (*p* < 0.001). The MDTA increased from 85.8 ± 2.8° to 91.7 ± 1.9°, indicating effective correction of coronal alignment. Clinically, the mean AOFAS score improved from 62.9 ± 6.6 to 87.1 ± 5.2, and the AOS pain score from 54.8 ± 6.5 to 28.9 ± 5.1 (both *p* < 0.001), confirming the functional and radiographic benefits of combining fibular and distal tibial osteotomy.

### 4.4. Plafond-Plasty

Although medial opening-wedge and lateral closing-wedge SMOTs have shown satisfactory results, as described above, residual intra-articular deformity is associated with a risk of failure. In 2009, Becker and Myerson first described a medial opening-wedge osteotomy of the tibial plafond (plafond-plasty) [14].

**Figure 4:**
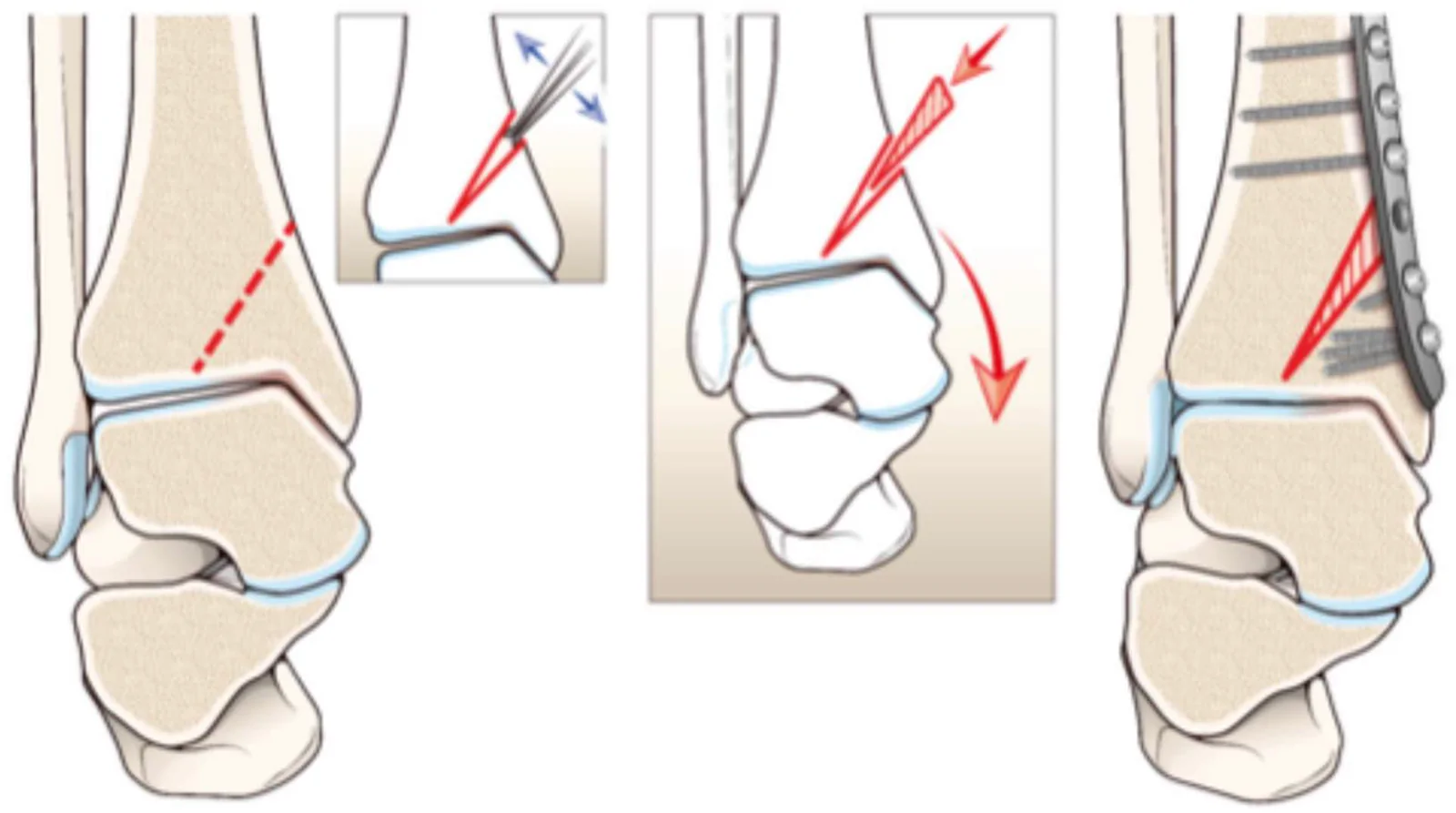
Illustration of plafond-plasty with bone graft (red arrow) for the correction of non-congruent ankle varus deformity.

Through a medial approach, a K-wire is used to guide the osteotomy plane toward the apex of the deformity, and three additional K-wires are inserted parallel to the articular surface of the tibial plafond, just beneath the articular cartilage. These wires prevent the saw from penetrating the joint and, together with the intact distal subchondral bone bridge, act as a hinge during correction. The plafond is then gradually repositioned with a wide osteotome until the medial articular surface is aligned with the intact lateral portion of the distal tibia. A lamina spreader is inserted into the gap to maintain the correction while the defect is filled with allograft bone chips [14,38]. Alternatively, a Hintermann distractor can be used to open the osteotomy progressively until the planned correction is achieved [39]. The osteotomy is then fixed with a locking plate (Figure 4).

The indications for this technique remain controversial, as highlighted by a recent systematic review by Zielli et al. [39]. Plafond-plasty has been described mainly for reducible intra-articular varus deformities, identified by correction under manual stress on fluoroscopy. Hintermann further quantified this criterion as a residual varus of 2° and defined an intra-articular deformity as an angular deviation greater than 5° [30,40]. Several authors have reported significant clinical and radiographic improvement after plafond-plasty. Guo et al. documented a marked reduction in VAS pain score (from 6.7 ± 0.8 to 3.1 ± 0.6) and an improvement in MDTA from 87.1 ± 1.5° to 92.5 ± 0.7° [41]. Similarly, Xu et al. observed a significant increase in AOFAS score from 47.7 ± 15.7 to 75.8 ± 12.0, with a reduction in TTS from 14.3 ± 5.0° to 5.3 ± 4.0° [42]. Regarding complications, Ma et al. reported hinge fractures in 15.8% of cases, which are potentially serious because of their intra-articular nature [43]. Guo et al. reported no intraoperative complications, although 23.2% of patients required additional surgical procedures [41].

### 4.5. Mortise-Plasty Osteotomy

**Figure 5:**
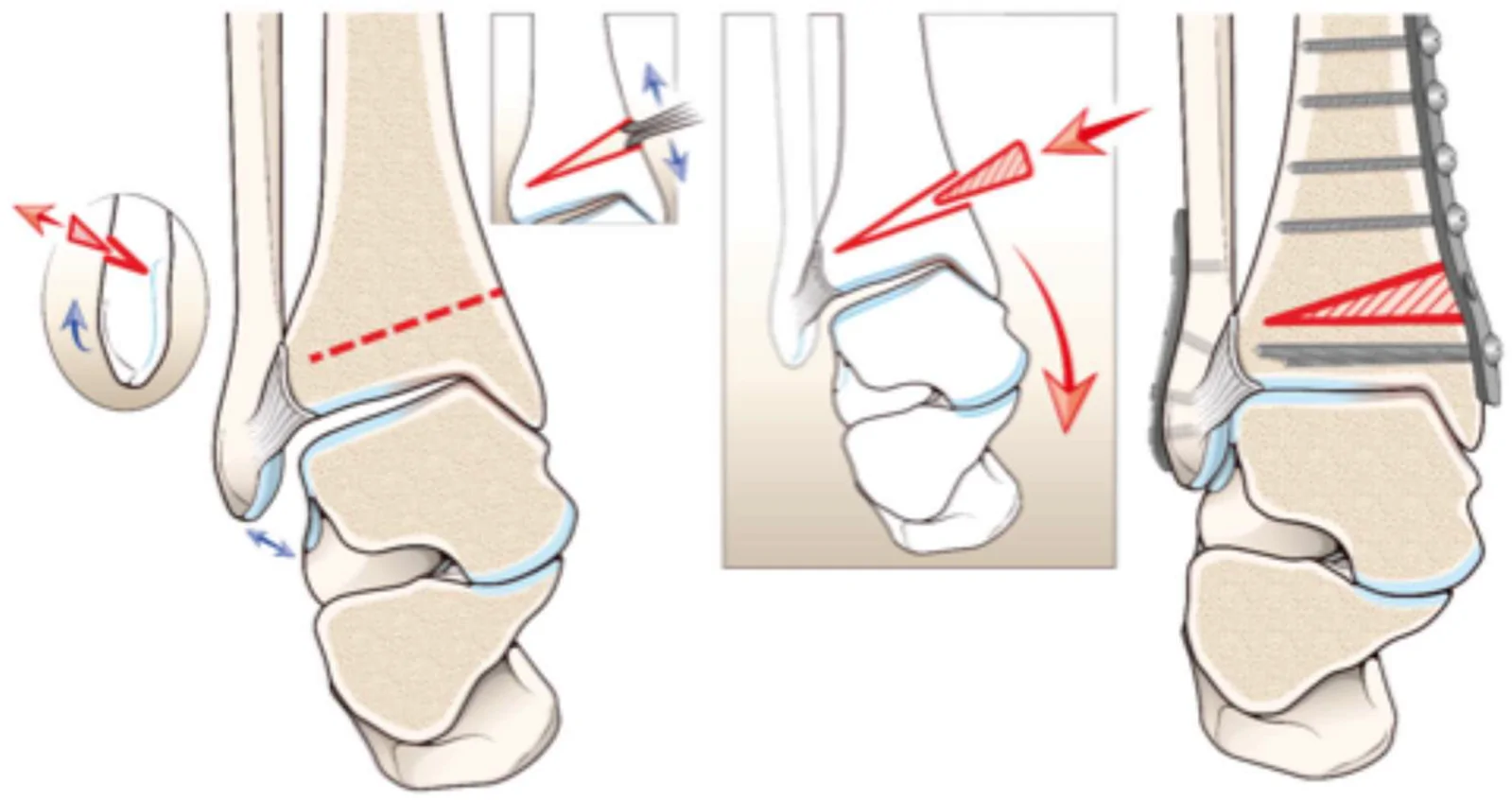
Illustration of mortise-plasty osteotomy with bone graft (red arrow) for the correction of non-congruent ankle varus deformity.

Kobayashi et al. described mortise-plasty osteotomy for varus ankle OA with a talar tilt that was reducible on valgus stress testing. In their original series, the procedure was performed in ankles with a talar tilt of at least 3°; this threshold should be regarded as a technical selection criterion used by the authors rather than as a universally validated indication [44]. A horizontal osteotomy was performed in the distal tibia 1.5 cm above the articular surface, to minimize the opening of the osteotomy and to bring it closer to the CORA in mixed deformities. The gap was filled with two wedges of β-tricalcium phosphate or interconnected porous hydroxyapatite ceramic, and the osteotomy was fixed with dedicated anatomical plates (Figure 5).

The authors reported good to excellent clinical results in 85% of cases. Postoperatively, 23 of 27 patients (85.2%) had a talar tilt of less than 3°, with a significant reduction from 8.3 ± 5.3° to 1.8 ± 2.2°, and the MDTA was corrected from 84.9 ± 3.0° to 95.0 ± 3.9° [44].

### 4.6. Dome Osteotomy

A dome osteotomy is generally considered appropriate for congruent deformities, i.e., malalignment in which the tibial plafond and talar dome remain parallel and congruent. In symmetric deformities, a dome osteotomy allows correction even when the CORA is not located exactly in the tibial metaphysis, without translating the load-bearing axis [45,46].

Through an anterior approach, a locking plate is prepared with drill sleeves fixed in both end holes. The center of one sleeve is aligned with the intersection of the mid-diaphyseal line of the tibia and a perpendicular line bisecting the distal tibial plafond. A Steinmann pin is inserted through this sleeve, parallel to the axial and sagittal planes and perpendicular to the coronal plane, so that the plate can rotate only in the coronal plane while sagittal movement is controlled. The osteotomy is performed within the metaphyseal bone, to promote union, by connecting multiple drill holes with an osteotome. After a fibular osteotomy, the distal tibial fragment is rotated to correct the deformity and fixed with plates and screws [47].

Faict et al. recently reported the clinical and radiographic outcomes of five patients with symmetric varus deformity without talar tilt treated with dome osteotomy using patient-specific cutting guides [48]. At a mean follow-up of 40.8 months (range, 8–65), the FAOS improved significantly from 32.2 to 65.2. The TAS angle also improved significantly, from 84.2 ± 9.34° to 93.00 ± 4.18°, resulting in slight overcorrection into valgus. No complications or failures were reported at final follow-up. However, the management of complex lower-limb deformities remains highly debated, particularly when the mechanical axis of the limb deviates in the direction opposite to the ankle deformity [49].

**Figure 6:**
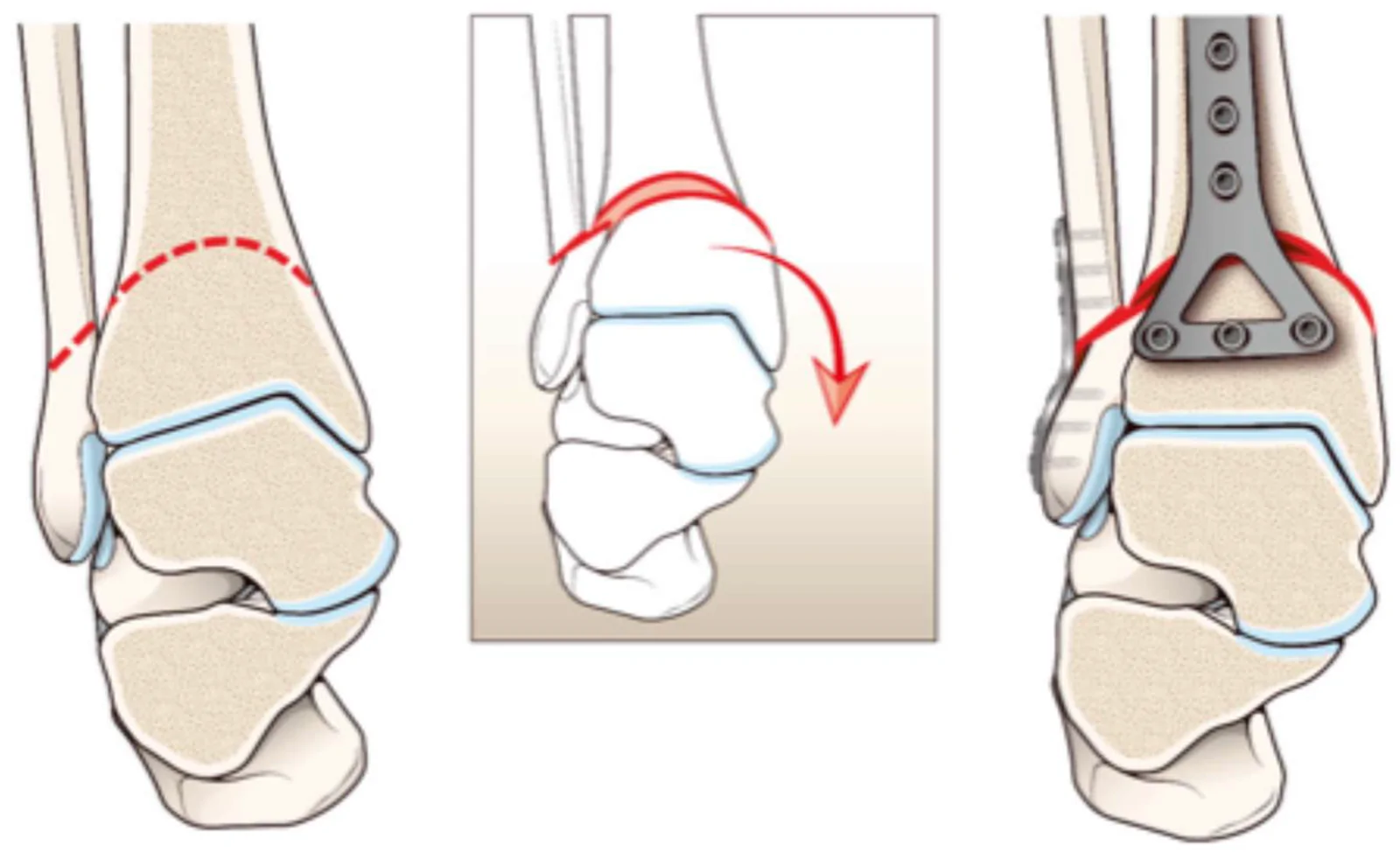
Illustration of dome procedure with fibular osteotomy and rotation (red arrow) for the correction of congruent ankle varus deformity.

In 2022, Kim et al. introduced a modified dome SMOT for patients with opposing coronal plane deformities of the ankle and the lower limb [50]. This technique allows simultaneous correction of distal tibial malalignment and of the lower-limb mechanical axis, avoiding the adverse translational effects often associated with conventional wedge osteotomies (Figure 6).

The mechanical axis of the whole lower limb was assessed using the hip–knee line to identify varus or valgus deformity, and patients with opposing deformities underwent the modified dome osteotomy. The tibial osteotomy was performed approximately 3 cm proximal to the ankle joint, just above the tibiofibular syndesmosis. The fibular osteotomy was performed first, followed by the tibial osteotomy, and alignment marks were drawn on the tibia as a reference for reduction. After the tibial osteotomy, the distal fragment was rotated and translated in the coronal plane. The T-plate was then reapplied and fixed with screws, and the fibular osteotomy was fixed with a one-third tubular plate. In patients with ankle varus and lower-limb valgus, postoperative TTS, MDTA, and TT improved significantly. Moreover, overall lower-limb alignment returned to neutral, with a lateral shift of the load-bearing axis that effectively unloaded the overloaded compartment.

The main characteristics, indications, advantages, limitations, possible role of fibular osteotomy, and specific complications of the techniques described above are summarized in Table 3.

## 5. Complications

SMOT is a reliable way to correct deformity in ankle OA, but it is technically demanding and carries a non-negligible complication rate.

A recent systematic review by Butler et al. analyzed 24 studies including 1160 patients who underwent SMOT for ankle deformity [10]. The overall complication rate was 5.1%, with nonunion being the most common (1.6%). Additional procedures were required in 28.2% of patients, most commonly hardware removal (17.6%). Specifically for varus deformity, Krähenbühl et al. reported a series of 44 patients treated with oblique medial opening-wedge osteotomy for post-traumatic asymmetric varus ankle OA [6,9]. Complications requiring further surgery occurred in 9% of cases, including three revisions for malunion or nonunion. The 5-year survival rate was 75% and was higher in ankles with a lower Takakura–Tanaka stage: ankles with preoperative stage 2 or 3A had significantly higher 5-year survival (88% [95% CI, 67–100] and 93% [95% CI, 80–100], respectively) than those with stage 3B (47% [95% CI, 26–86]) [6]. In a small series of nine patients treated with lateral closing-wedge osteotomy, Harstall et al. reported no complications and a mean healing time of 10 weeks (range, 6–14; SD ± 3.31) [35]. The main complications, risks, and pitfalls of SMOT are summarized in Table 4.

Technique-specific risks should also be considered. Medial opening-wedge osteotomy may be complicated by lateral hinge fracture, loss of correction, and medial soft-tissue irritation, whereas lateral closing-wedge procedures carry the risks associated with fibular osteotomy, including delayed union or nonunion and lateral soft-tissue or nerve irritation. Intra-articular procedures such as plafond-plasty carry an additional risk of intra-articular hinge fracture: Ma et al. reported three hinge fractures in 19 patients (15.8%), although all osteotomies eventually healed [43].

### Patient-Specific Instrumentation and 3D Planning

Recent advances have highlighted the role of patient-specific instrumentation (PSI) and 3D planning in improving the accuracy and reproducibility of SMOT. PSI allows precise preoperative planning of the osteotomy location and correction angles based on 3D reconstructions and the production of custom cutting guides. This approach has been shown to reduce intraoperative fluoroscopy time, lower the risk of technical errors, and facilitate achievement of the planned alignment, particularly in complex multiplanar deformities [51].

In varus ankle OA, a two-step 3D-guided technique combining detailed virtual planning with intraoperative patient-specific guides has shown high accuracy of correction and improved reproducibility, even in the presence of advanced degenerative changes. These technologies are particularly valuable in challenging scenarios, such as post-traumatic deformities after previous fractures, in which rotational abnormalities and malalignment of the entire lower limb coexist. Overall, these advances may contribute to more reliable corrections and potentially better clinical outcomes [52,53].

## 6. Discussion

**Figure 7:**
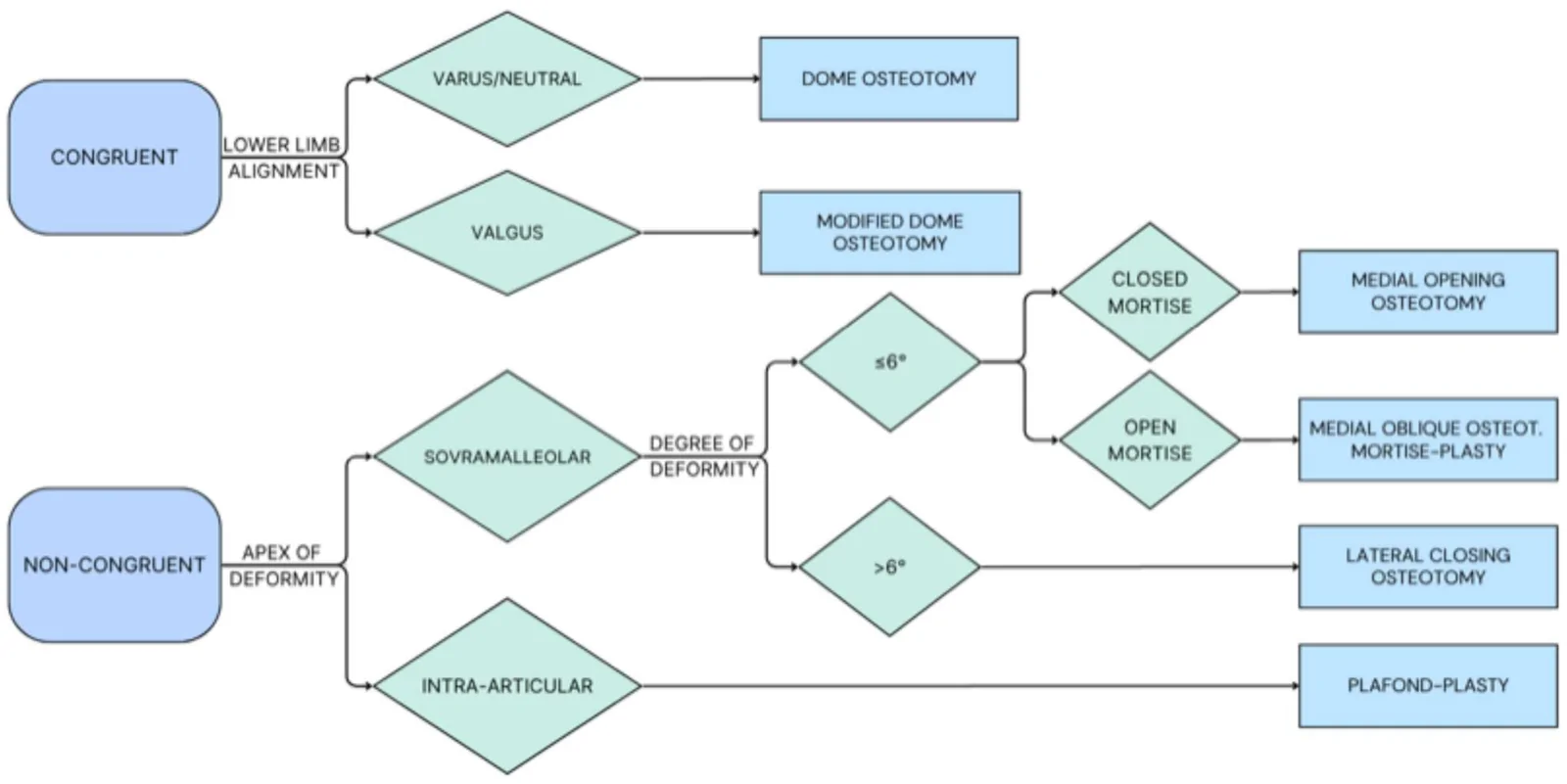
Author’s algorithm of treatment for SMOT in varus ankle.

We propose a structured treatment algorithm for SMOT in varus ankle OA (Figure 7).

This algorithm reflects the authors’ expert opinion, informed by the available literature, and should be regarded as a practical guide rather than a set of universally validated recommendations. The framework incorporates the Takakura–Tanaka stage, joint congruency and talar tilt, the location of the CORA, the magnitude and level of the deformity, and the overall lower-limb mechanical axis to guide the choice between joint-preserving and joint-sacrificing treatment.

In congruent OA, dome osteotomy is generally preferred, as it allows multiplanar correction, including coronal deformity, without translating the mechanical load axis [50]. In particular, when opposing deformities are present, such as ankle varus combined with valgus alignment of the lower limb, the modified dome osteotomy described by Kim et al. offers an effective solution, allowing precise local correction while restoring overall limb alignment [50].

In incongruent OA, the primary location of the deformity determines the surgical strategy. For intra-articular deformities, plafond-plasty is recommended despite its higher risk of intra-articular fracture, as it remains the most effective way to restore joint congruency. For supramalleolar deformities, the magnitude of angular deformity is a key factor in choosing the approach. For larger supramalleolar deformities, typically exceeding approximately 10°, a lateral closing-wedge osteotomy may be considered when a medial opening wedge would require substantial grafting or place excessive tension on the medial soft tissues. This approach also allows a concomitant fibular osteotomy through the same lateral incision, which may facilitate talar reduction and improve mortise congruency; the lateral closing-wedge osteotomy therefore addresses both the bony deformity and the fibular constraint through a single incision. Smaller deformities are usually treated with a medial opening-wedge osteotomy, favored for its technical simplicity and the possibility of gradual, controlled correction [33,54]. When the required correction exceeds approximately 6°, a concomitant fibular osteotomy may be considered, as it may facilitate talar translation and improve correction of joint incongruence by promoting a more even distribution of contact pressure.

In cases associated with tibiofibular mortise instability, a medial transverse osteotomy or a mortise-plasty osteotomy may be recommended, depending on the degree of talar tilt. Fibular osteotomy also plays a key role, as it allows slight overcorrection and facilitates correction of any concomitant intra-articular deformity.

When both supramalleolar and intra-articular deformities are present, we recommend a sequential approach: first, correction of the supramalleolar component to restore limb alignment; second, targeted management of the intra-articular deformity according to the “four-column theory,” an original concept proposed by the authors. This concept identifies four structures that contribute to persistent intra-articular malalignment and can be selectively addressed to optimize correction: the lateral malleolus, the medial malleolus, the lateral ligament complex, and the deltoid ligament complex. According to this model, each column may act either as a source of deformity or as a target for surgical correction. By systematically assessing and addressing these components, the surgeon can more effectively restore joint congruency and stability.

Soft-tissue balancing is essential for successful correction but is regarded as an adjunct to accurate bony realignment. When correcting varus deformity, lateral capsuloligamentous reconstruction and/or deltoid ligament release are recommended to optimize soft-tissue tension. However, soft-tissue procedures alone cannot prevent recurrence of coronal deformity if bony malalignment persists; they should therefore be performed only after bony correction has been completed, to ensure long-term stability and alignment [14].

Regarding the choice of osteotomy, supramalleolar opening- and closing-wedge osteotomies have shown promising results in correcting extra-articular varus deformity by lateralizing the load-bearing axis. However, they do not address concomitant intra-articular deformity unless combined with procedures targeting the four columns. The oblique osteotomy at the level of the syndesmosis has shown good corrective power and a mortise-closing effect, but outcomes tend to be less favorable when talar tilt exceeds 5–7°. In such cases, adding a Z-shaped fibular osteotomy at the same level has been associated with better results and more reliable reduction in talar tilt [38].

Overall, SMOT for varus ankle OA should be regarded as a joint-preserving procedure aimed at delaying, for as long as possible, the need for further surgery such as TAR or AA. However, given the poorer outcomes reported in advanced OA and the limited corrective capacity of these techniques, which are themselves associated with inferior results in the presence of large deformities, the authors believe that joint-preserving surgery is not always advisable [3,55]. Specifically, in advanced OA (Takakura–Tanaka stage ≥3B) without severe varus deformity (talar tilt <10° and tibiotalar varus angle <10–15°), TAR should be considered. Conversely, in advanced OA (Takakura–Tanaka stage ≥3B) with marked varus deformity (talar tilt >10° and tibiotalar varus angle >10–15°), the authors consider AA to be the most appropriate option.

## 7. Conclusions

OA of the tibiotalar joint remains one of the most common and challenging conditions in foot and ankle surgery. SMOT is a reliable joint-preserving option for patients with malalignment. Optimal outcomes require meticulous preoperative evaluation and planning, including appropriate patient selection and accurate classification of the deformity through clinical and radiographic assessment; precise measurement of key radiographic parameters to identify the CORA and to evaluate mortise stability and lower-limb alignment; and careful surgical planning to determine the required correction and the need for adjunctive procedures. Finally, selecting the most appropriate technique, tailored to each patient’s anatomy and risk profile, is essential to restore alignment, relieve pain, and preserve joint function.

## Figures and Tables

**Table 1:** Main indication and contraindication for SMOT procedure.

Indications	Congruent ankle OA associated with varus deformity Incongruent medial ankle OA with laterally preserved cartilage with mild deformity (talar tilt < 10 degrees and tibiotalar varus angle < 10–15 degrees) Takakura–Tanaka stage ≤ 3A
Contraindications	End-stage OA (Takakura 4) Unmanageable ankle instability/ neuromuscular imbalance Osteomyelitis or infection Severe vascular or neurological deficiency
Relative Contraindications	End-stage OA (Takakura 3b) Tobacco use Diabetes Mellitus Osteoporosis Altered bone quality Age (>60 years)

**Table 2:** Anatomical classification of apex’s deformity in varus ankle osteoarthritis.

TAS	TTS	TT	DEFORMITY
<93.3 ± 3.2°	<87.2 ± 2.8°	<4°	Supramalleolar
93.3 ± 3.2°	<87.2 ± 2.8°	>4°	Intrarticular
<93.3 ± 3.2°	<87.2 ± 2.8°	>4°	Mixed

**Table 3:** Summary of osteotomy options for varus ankle osteoarthritis.

OSTEOTOMY	MAIN INDICATION	MAIN ADVANTAGE	FIBULAR OSTEOTOMY	LIMITATION/PITFALLS
**Medial opening-wedge**	Mild-to-moderate supramalleolar varus	Gradual, adjustable correction	Selective	Medial soft-tissue tension; graft may be needed
**Lateral closing-wedge**	Larger supramalleolar varus	Stable correction; no opening-gap graft	Often combined; same lateral approach	Limb shortening; less adjustable correction
**Oblique distal tibial osteotomy**	Varus with medial mortise widening and limited talar tilt	Corrects varus and narrows mortise	Consider if talar reduction is limited	reduction is limited Less predictable with marked talar tilt
**Plafond-plasty**	Reducible intra-articular varus deformity	Restores articular congruency	Selective	Intra-articular hinge fracture
**Mortise-plasty**	Mortise instability with reducible talar tilt	Corrects deformity close to CORA; improves mortise congruency	Usually not required	Limited evidence; correction limits unclear
**Dome osteotomy**	Congruent or complex/multiplanar deformity	Multiplanar correction without major translation	Often required	Technically demanding

**Table 4:** Main complications, risks and pitfalls.

Neurovascular or tendon injury
Wound-healing problems, infection, or soft-tissue irritation
Overcorrection or under-correction
Loss of correction with osteoarthritis progression
Malunion/nonunion
Symptomatic hardware requiring implant removal

## Data Availability

No new data were created or analyzed in this study. Data sharing is not applicable to this article.
